# The Management of Sacral Schwannoma: Report of Four Cases and Review of Literature

**DOI:** 10.1155/2008/845132

**Published:** 2008-09-02

**Authors:** Chandhanarat Chandhanayingyong, Apichat Asavamongkolkul, Nittaya Lektrakul, Sorranart Muangsomboon

**Affiliations:** ^1^Department of Orthopaedic Surgery, Faculty of Medicine Siriraj Hospital, Mahidol University, 2 Prannok road, Bangkoknoi, Bangkok 10700, Thailand; ^2^Department of Radiology, Faculty of Medicine Siriraj Hospital, Mahidol University, 2 Prannok road, Bangkoknoi, Bangkok 10700, Thailand; ^3^Department of Pathology, Faculty of Medicine Siriraj Hospital, Mahidol University, 2 Prannok road, Bangkoknoi, Bangkok 10700, Thailand

## Abstract

Sacral schwannoma is a rare retrorectal tumor in adults. Postoperative sacral neurological deficit is difficult to avoid. Currently, there is no established consensus regarding best treatment options. We present the management and outcomes of sacral schwannoma in 4 patients treated with intralesional curettage and postoperative radiation. There were 3 women and one man (average age: 45.5 years) with long duration of lumbosacral pain with or without radiculopathy. Intralesional curettage was performed by posterior approach and adjuvant radiation therapy with dosage of 5000–6600 cGy was given after surgery. The mean follow-up time was 18 months (range 4–23 months). Symptoms of radiculopathy had decreased in all patients. The recent radiographic findings show evidence of sclerosis at the sacrum one year postoperatively, but the size was unchanged. Intralesional curettage and adjuvant radiation therapy can be used in the treatment of sacral schwannoma to relieve symptoms and preserve neurological function.

## 1. INTRODUCTION

Schwannoma is a benign neoplasm of schwann cell, arising along sensory nerve roots in the extremities and upper thorax. These tumors rarely arise within bone, among which mandible and sacrum are the most common sites of involvement. Only 79 intraosseous schwannomas have been reported in English literature and 21 were located at the sacrum [1]. Most of the cases were treated by curettage and overall results were favorable due to preservation of sacral nerve roots [1–14]. However, the rate of local recurrence was reported to be relatively high (54 percent) when treated by conservative means. Abernathey et al. suggested wide excision of sacral schwannoma to prevent tumor recurrence [6]. Many authors reported that sacral amputation and lumbopelvic fixation allowed total removal of sacral schwannoma [4, 6–14]. The patients who were treated with sacral amputation had greater chance of having postoperative bowel and bladder dysfunction, in addition to decreased sensation and motor weakness of lower extremities due to sacral nerve roots injury. Excision of the tumor might cause extensive blood loss from combined anterior and posterior approaches. The surgical technique is needed and instrumentation is required to maintain spinal stability. Even though attempts to minimize such complications were done by laparoscopic or gamma knife surgery, the results are still in their study periods and only a limited number of cases were included [15–19]. We report a series of 4 cases of sacral schwannoma treated by intralesional curettage and postoperative radiation therapy. Relatively conservative methods were used due to the benign condition of the tumor. The clinical outcomes and sequential radiographic results are presented.

## 2. PATIENTS AND METHODS

Between July 2005 and March 2008, three women and one man with sacral schwannoma were treated in our institute. Mean age of the patients was 45.5 years (range 29–62 years). All patients presented with lumbosacral pain, of duration from 8 months to six years (mean 3.4 years). Three patients complained of pain that later progressed to sciatica and dysethesia of one or both legs. One of them developed difficulty in urination and also had constipation. Neurological examination of patient no. 2 revealed no lower extremity weakness, but decreased deep tendon reflex of the left ankle joint, and diminished left anterior thigh and perianal sensation. The other three patients present no neurological deficit except a loss of sensation in the left great toe in patient no. 3 (Table 1).

**Figure 1:**
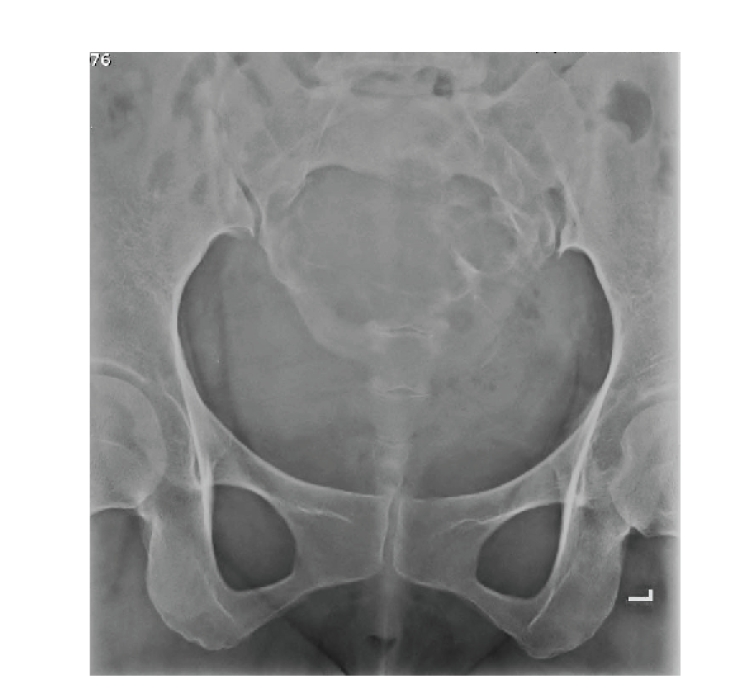
Plain radiograph shows osteolytic bony destruction at body of sacrum and bilateral neural foramens.

**Figure 2:**
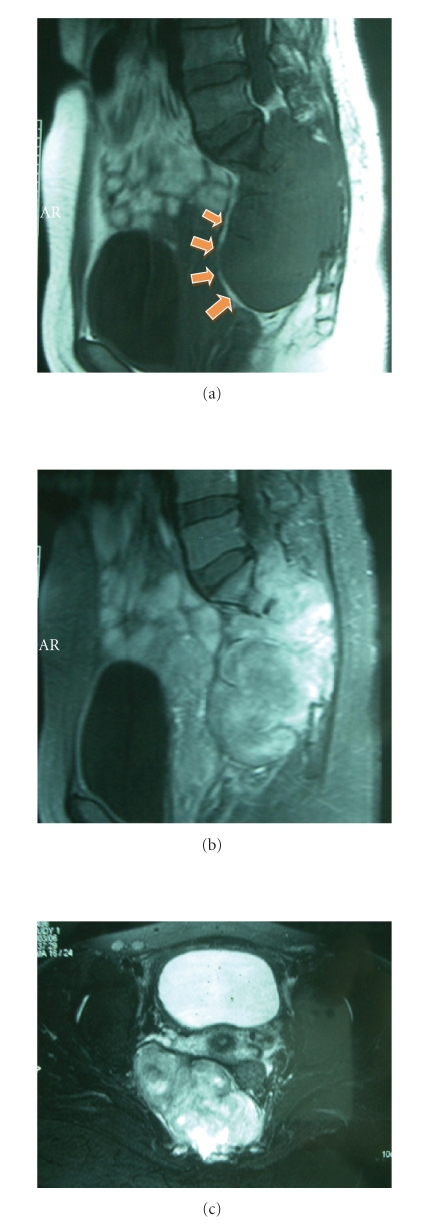
MR examination of pelvis demonstrates bony destruction at sacrum and neural foramen from S1–S4 levels with soft tissue mass formation. This mass appears as low signal intensity on sagittal T1-weighted MR image (Figure 2(a)), heterogeneous increased signal intensity on sagittal and axial T2-weighted MR image (Figures 2(b) and 2(c)). Tumor extended and displaced uterus and urinary bladder anteriorly, but not invaded, evidenced by fat plane (arrows).

**Figure 3:**
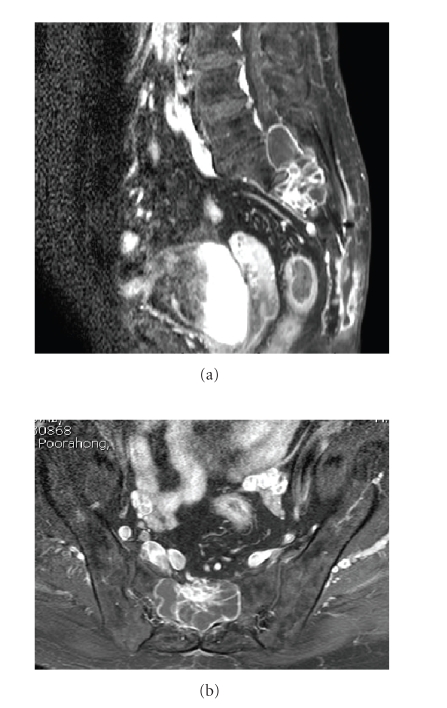
Sagittal (Figure 3(a)) and axial (Figure 3(b)) T1-weighted/GD/FS MR images show a well-defined extradural cystic mass with multiple enhanced septations and thin enhanced solid nodule, originating from the posterior aspect of S1–S3 region.

**Figure 4:**
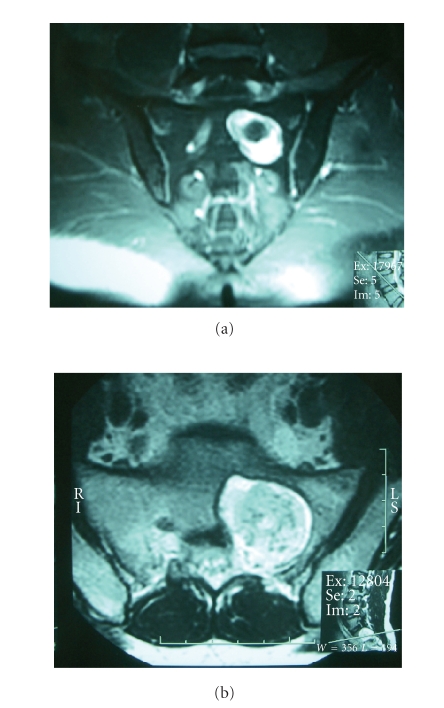
Coronal (Figure 4(a)) and axial (Figure 4(b)) T2-weighted MR images show tumor with heterogeneous signal intensity involving left S1 foramen.

**Figure 5:**
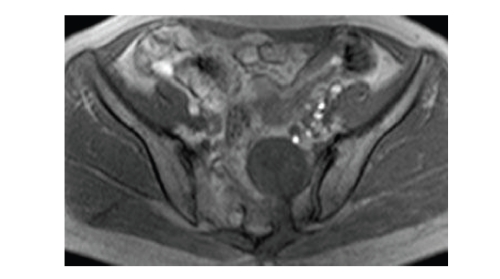
Axial T1-weighted MR image demonstrates dumbbell-shaped tumor which arising from sacral foramen and extended anteriorly to pelvic cavity in patient no. 4.

Plain radiographic findings showed an extensive osteolytic lesion with sclerotic border which involved the whole sacrum of two patients (nos. 2 and 4) (Figure 1) and only an ill-defined osteolytic lesion mainly occupying the left side of S2-3 area in the other two patients (nos. 1 and 3). MRI revealed iso-intensity to hypointensity imaging on T1-weighted sequence and hyperintensity imaging on T2 weighted in all patients. Mean maximal length of the tumor was 8.1 cm (range 4.4–11.0 cm) and mean volume was 259.8 cm^3^ (range 50.6–770.0 cm^3^). The tumor extended into surrounding tissue and displaced abdominal structures anteriorly in two patients (nos. 2, 4), but the fat plain between tumor and abdominal cavity was evidenced in all cases (Figure 2). This finding indicated that there was no tumor invasion into internal organs. In one patient (no. 1), the tumor expanded only posteriorly and compressed the dural sac, with multicystic lesions (Figure 3). All MRI findings demonstrated tumor extension from neural foramen (Figure 4), and one was seen as a dumbbell-shaped configuration (Figure 5). This appearance clarified by tumor arising within the sacral foramina as the narrowest part and expanded intrasacral and displaced pelvic organs anteriorly, which could be seen in axial MR imaging.

In all patients, intralesional curettage was performed by posterior approach through sacral laminectomy. The tumor capsule remained intact. Two tumors were intrasacrally confined and 2 extended extrasacrally, but all were extradural. Intraoperative nerve stimulators were not performed. After tumor removal, lumbosacral and sacroiliac stability of all patients was not changed. No reconstruction and instrumentation was performed in any patients. Histological finding showed typical schwannoma in all 4 cases. There was no appearance of degenerated neurilemmoma (ancient schwannoma) or hypercellular tumor (cellular schwannoma) in this series.

After the wounds had healed at six to eight weeks, radiation therapy was performed in the out-patient clinic using dosage of 5000–6600 cGy.

## 3. RESULTS

At the mean follow-up time of 18 months, all patients experienced relief of lumbosacral and radicular pain after surgery. Urinary hesitancy was improved in patient no. 2. Perianal sensation was subsequently improved at six months postoperatively. No neurological worsening occurred in any patients. No recurrent symptoms were evidenced afterward. The patients could walk well without gait aids.

**Figure 6:**
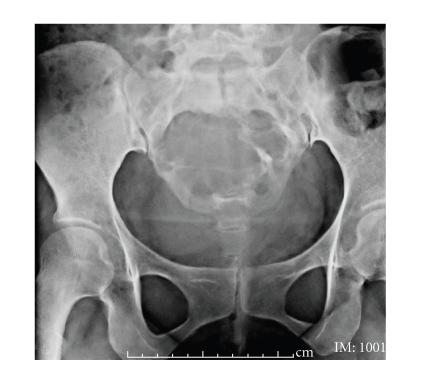
Plain radiograph, 1 year post operative shows sclerosis of bony destruction at sacrum. MR examination of pelvis one year after surgery demonstrates no significant change in size and aggressiveness of the tumor, but not increase.

Plain radiographs showed marginal sclerosis at the lesion and destruction of the sacrum had not progressed (Figure 6). Only patient no. 2 showed a nondisplaced fracture of S2 on the left side as a result of preoperative massive bony damage. MRIs were performed two months after surgery and revealed that tumors were eradiated resulting as cystic portion, but two patients demonstrated small amount of residual tumor (Table 1). The presacral cysts were persistent in the same size as before surgery. Although the cystic lesion did not disappear, its progress was stopped by radiation.

Patient no. 2 had amenorrhea permanently after radiation. She was counseled earlier and decided to have radiation therapy due to the large size of the tumor and a postoperative residual tumor that was evidenced through MRI.

## 4. DISCUSSION

Of all malignant tumors originating in sacrum, chordomas, chondrosarcoma, and metastatic lesions are most frequently observed. Benign sacral lesions including giant cell tumor, aneurysmal bone cyst, and osteoblastomas are occasionally evidenced whereas schwannomas are very rare [20]. There is no sex predilection in this tumor [1, 4, 6, 13, 21, 22]. although in our series male female ratio was 1:3. Lumbosacral pain with or without radiculopathy were the most frequent symptoms noted in previous studies and in our patients. Decrease in lower extremity sensation is the most frequent neurological sign found. A tumor which was confined in a more proximal spinal level would cause early seeking of medication and result in earlier detection than a tumor which is contained in the lower spinal level [3, 13]. In the same way, tumor extension posteriorly to dural sac and nerve roots would be earlier symptomatic than a tumor which extends anteriorly adjacent to abdominal structure.

Plain radiograph of the lumbosacral area often fail to be noticed especially when the lesion is undersize. CT is useful to detect degree of bony destruction, but an MRI provides a better display in multiple views of the sacral mass. In all of our patients, MRI were performed and demonstrated details of intrasacral, intrapelvic, intra- or extradural, and nerve root compression, as well as displaying the relationship to neighboring structures. This information aids in preoperative diagnosis and surgical management.

Many studies in surgical treatment for sacral schwannoma have been reported. Abernathey et al. reported 13 cases of schwannoma of the sacrum [6]. Nine patients in this study who were treated by intralesional curettage experienced tumor recurrence and underwent additional surgery (54 percent) with follow-up periods ranging from 5 months to 33 years (mean 9 years). Their study suggested that schwannoma originating in the sacrum should be aggressively resected with the aim of complete extirpation and that sacrifice of all or many nerve roots was required to minimize the risk of recurrence. In contrast, Dominguez et al. reassured us that a conservative approach with intracapsular enucleation alone produced a favorable result of only 16 percent recurrence rate [1]. The follow-up period was range from 18 months to 21 years (average 9.2 years). The follow-up time of our series might be too early to conclude that, though we used a conservative approach, the recurrence rate was 0 percent with a follow-up period of 7 months to 27 months (mean 18 months).

Role of adjuvant radiation therapy is controversial. Kotoura et al. presented one case that was treated with intralesional curettage and adjuvant radiation therapy [23]. The attempt was made to preserve nerve roots as much as possible. The patient was followed up for 5 years, and plain radiograph and CT scan showed arrest of the tumor with marginal sclerosis. However, Feldenzer et al. revealed that the tumor did not respond at all [24]. Conventionally, radiation therapy is avoided in the treatment of benign tumors, because of the risk of secondary carcinogenesis [25]. However, radiation therapy is obligatory in some cases in which anatomic location of the tumor does not allow total extirpation, or in which aggressive resection may cause serious functional damage. It has been reported that cases of giant cell tumor at the lumbosacral area can be controlled by radiotherapy [26–28].

For benign tumors at this particular location, the patients have minimal risk of distant metastasis, low rate of recurrence, and excellent prognosis. It is desirable to preserve the functions of lower extremities, as well as bowel and bladder function after the treatment.

## 5. CONCLUSIONS

This is a report of a rare clinical entity. Although the number of the patients and the length of follow-up are limited, we made the conclusions as the followings. Clinical courses were longer in the patient who had anterior tumor extension and lower level of spinal involvement (average 4.3 years) than posterior extension and upper level of spinal involvement (8 months) which compress the dural sac and sacral nerve root more rapidly. But when compared to tumors in other regions, the duration is still extremely long. The symptoms of radicular pain, loss of motor power, decreased sensation of lower extremities, and bowel bladder symptoms would subsequently occur after time. MRI is an important diagnostic tool because plain X-ray films of the lumbosacral area are often inconclusive. The tumors always originate from one side of the sacral foramen and extend to the adjacent structures, and are recognized as having a dumbbell shape. This finding is useful for preoperative diagnosis. Multicystic appearance and small foci of calcification or residual bone may be evidenced in some tumor. No tumor has infiltrated to the peritoneum and intra-abdominal organs, as diagnosed before surgery by appearance of the fat plane. A conservative approach by intralesional curettage and radiation is advised in view of the benign course of the disease. All patients had improvement in symptoms and none of them experienced clinical worsening after surgery. Recurrence has not been evidenced in this study. One female patient had permanent amenorrhea after radiation. The treatment by high-dose postoperative radiation must be judged with regard to risks and benefits especially in women of reproductive age. Further follow-up studies must be conducted.

## Figures and Tables

**Table 1:** Clinical Summary of the patients.

	Age/sex	Symptoms/duration	MRI	F/U time (months)	Follow-up
Case 1	62 F	Sacral pain, radiates to rectal vault/8 months	Multicystic lesions of S1-2, posteriorly invaded extradural, adjacent to thecal sac	27	Asymptomatic, no recurrence on MRI
Case 2	29 F	Lumbosacral pain, radiculopathy, and decrease sensation of Rt. leg and Lt. thigh, decrease perianal sensation, urinary hesitancy, constipation/5 years	Solid-cystic lesions of S2-4, anterior extension	21	Asymptomatic in lumbosacral and radicular pain, subsequent improvement in urinary function, incomplete improvement on perianal sensation, amenorrhea, small residual tumor on MRI, nondisplaced fracture S1
Case 3	39 M	Lumbosacral pain, radiculopathy of Lt. leg, loss sensation of Lt. great toe/2 years	Solid dumbbell shape mass in S1-2, intrasacral	18	Asymptomatic, no recurrence on MRI
Case 4	52 F	Lumbosacral pain, radicular pain of Rt. leg/6 years	Solid mass of S1-2, anterior extension	7	Asymptomatic, small residual tumor with seroma on MRI
